# Exploring Psycho-Social Factors Influencing Exclusive Breastfeeding: Lived Experiences of First Time Mothers at Salaga Municipal Hospital, Savannah Region in Ghana

**DOI:** 10.3389/ijph.2025.1607542

**Published:** 2025-01-30

**Authors:** Felix Amekpor, Christa Osei-Mensah, Nancy Adjeley Adjieteh, Esther Nyaapoka Dok-Yen, Memunatu Babariyor Salamu

**Affiliations:** ^1^ Noguchi Memorial Institute for Medical Research, University of Ghana, Accra, Ghana; ^2^ Department of Physician Assistantship Studies, Garden City University College, Kumasi, Ghana; ^3^ Catholic Primary Health Care -Salaga, Bolgatanga, Ghana; ^4^ Shishegu Community Health Based Planning and Services, Tamale, Ghana

**Keywords:** psychosocial, exclusive breastfeeding, infant and young child feeding, mothercare, lived experiences

## Abstract

**Objectives:**

Exclusive breastfeeding is a critical public health strategy known to reduce infant mortality and morbidity, and to enhance maternal and child health outcomes. This study explores the psychosocial factors affecting exclusive breastfeeding (EBF) among first-time mothers in Ghana, highlighting the challenges faced by first-time mothers in maintaining EBF for the recommended 6 months.

**Methods:**

The study utilized an exploratory qualitative research design to gain in-depth understanding of first-time mothers’ experiences with EBF, utilizing semi-structured interviews at the Salaga Municipal Hospital postnatal clinic.

**Results:**

The study found stressors affecting mothers’ exclusive breastfeeding practices, including lactation issues, infant positioning issues, and physical discomfort. Strong family support was found to be crucial for encouraging and sustaining EBF practices.

**Conclusion:**

Psychosocial factors significantly influence exclusive breastfeeding among first-time mothers. Strategies include addressing lactation issues, providing infant positioning education, alleviating physical discomfort, and enhancing family support systems.

## Introduction

Global health departments advocate the practice of exclusively breastfeeding at the initial stages of an infant’s life since it helps stimulate and enhances the development of the mouth and jaws cells in babies and ensures the growth of major organs in newborns [1]. It aids in brain development and enhances the intellectual capacity of the child. This feeding practice helps build the immune system and protects the baby against diseases [1]. According to a statement by the World Health Organization (WHO), “Breastfeeding is the cornerstone for an infant’s survival, nutrition and development” [2]. Early initiation of breastfeeding and exclusive breastfeeding help in child survival, it accounts for healthy brain development, promotes cognitive and sensory performance and is noted for enhancing intelligence and academic performance in children [3]. Feeding an infant with only breast milk is advocated by stakeholders in health, is one of the most important practices in an infant’s life and the best way a mother can invest into the wellbeing of her child.

Among the numerous benefits of breastfeeding, UNICEF in a breastfeeding Campaign in 2013, termed the essence of breastfeeding as a “first immunization and an inexpensive life saver.” Exclusive breastfeeding is acknowledged as the optimal way to feed infants for the first 6 months by national and many other health organizations [4, 5].

In low income and developing countries, due to poor sanitation conditions, high disease burden and limitedness in the availability of clean drinking water, it is more necessary to practice exclusive breastfeeding in the initial stages in life (first 6 months of the child’s life). It has been reported in several articles on breastfeeding that proper practice of breastfeeding can save about 800,000 infant lives in the developing world alone [6]. In spite of these recommendations, it has been documented over the years that the practice of exclusive breastfeeding has not been adopted universally, most mothers embrace the idea but fail to breastfeed exclusively few weeks after giving birth to their baby. Ghana’s exclusive breastfeeding rate stand currently at 43 percent, representing a significant drop from 63 percent in 2008. However, the target rates by WHO for exclusive breastfeeding 2030 are 70% [7]. Even in urban communities such as Tamale, the rate of EBF is as low as 27.7% [8] Breastfeeding exclusively will be much easier and attractive to mothers if the right health education, support and motivation are given [9]. An idea about the level of knowledge, attitude and practice of exclusive breastfeeding and the social support system available to mothers are very imperative for improvement in breastfeeding practices. It helps in reducing child mortality, promotes growth and immunity [10].

Factors, such as breastfeeding self-efficacy, dispositional optimism, and faith in breast milk, anxiety, depression and breastfeeding intentions are some common stresses among first time mothers [11]. The aim of this study was to assess the psychosocial factors that influence exclusive breastfeeding practice, the lived experiences of first-time mothers with infants between the ages of 0–6 months who attend post-natal clinic at the Salaga municipal hospital of the Savannah region of Ghana. By studying and bringing out how psycho-social factors influence the practice of exclusive among first-time to the fore, this study will serve as a basis for why family members should support the practice of exclusive breastfeeding among first time mothers. The study concluded that lactation problems, pain in the back and the nipple, positioning the baby to the breast were stressors for the mothers. The study also concluded that the society does not encourage women to practice exclusive breastfeeding.

## Methods

### Study Site

This study was conducted at the Salaga Municipal Hospital, located in the Salaga Municipality, Savannah part in Ghana. The hospital is a central healthcare facility providing maternal and child health services, making it an appropriate setting for exploring the psychosocial factors influencing exclusive breastfeeding among first-time mothers.

### Study Design and Participants

The study utilized a qualitative exploratory design to provide an in-depth understanding of the psychosocial factors affecting exclusive breastfeeding. Twelve first-time mothers who were currently breastfeeding and attending post-natal clinics at the hospital were selected using non-purposive sampling. Each participant was assigned a number to maintain confidentiality during analysis.

### Data Collection Methods

A semi-structured interview guide was developed and used to collect data. The guide consisted of open-ended and probing questions designed to elicit rich, detailed responses from participants. To ensure clarity and accuracy, the interview instrument was pre-tested on two first-time mothers in Kayereso, allowing the researchers to refine and eliminate ambiguities.

Individual interviews were conducted by the researchers at the hospital, lasting approximately 30–40 min each. Before the interviews, participants were briefed on the purpose of the study and provided written consent. The interviews were conducted in English and three local languages (Hausa, Gonja, and Twi), depending on the participant’s preference. All sessions were audio-recorded, transcribed, and translated where necessary. The data was subsequently coded from the recordings for analysis.

### Data Analysis

The coded data was systematically analyzed to identify emerging themes and patterns related to the psychosocial factors influencing exclusive breastfeeding. A thematic analysis approach was employed to ensure the findings were grounded in the participants’ experiences.

### Ethical Considerations

Ethical approval for this study was obtained from the Department of Midwifery at Garden City University College, Ashanti region, Ghana. Additionally, an introductory letter from the Dean of Students’ Office was submitted to the management of Salaga Municipal Hospital, Savannah region in Ghana, granting permission to conduct the research. All participants were informed about the study’s objectives and provided written consent. Anonymity and confidentiality were maintained throughout the research process.

## Results

This study included a total of 12 first-time mothers. Of the 12 participants, 3 were Christians and nine were Muslims. 4 participants had formal education and employed in the public sector whereas 8 were had basic education. In addition to working as housewives, the participants were also engaged in farming, trading, or hair dressing. The youngest mother was about 19 years old whilst the eldest was 35 years old. Seven of the participants were married whilst five were not married. Details of the demographic characteristics are shown in Table 1 below.

**TABLE 1:** Socio-demographic characteristics of participants (Savannah, Ghana. 2024).

Participants	Age	Level of education	Employment status	Religion	Marital status
Participant 1	31	Tertiary	Unemployed	Muslim	Married
Participant 2	25	Tertiary	Employed	Christian	Married
Participant 3	19	SHS	Farmer	Muslim	Single
Participant 4	30	Tertiary	Teacher	Muslim	Married
Participant 5	19	SHS	Trader	Muslim	Married
Participant 6	28	Tertiary	Nurse	Christian	Single
Participant 7	35	Primary	Trader	Christian	Married
Participant 8	19	JHS	Hairdresser	Muslim	Single
Participant 9	19	JHS	Unemployed	Muslim	Single
Participant 10	32	SHS	Teacher	Muslim	Married
Participant 11	19	JHS	Trader	Muslim	Single
Participant 12	26	SHS	Seamstress	Muslim	Married

### Mother’s Stressors

Study participants were asked to describe the personal and health problem they encounted that gave them stress with regards to breastfeeding.

#### Lactation Problems

Lactation problem was one of the major problems which was described by the participants. As first times mothers, most of them said they wanted to put their baby to breast immediately after birth but lactation was not established. One of the participants expressed that:

“*When I delivered, the breastmilk didn’t flow for me to breastfeed my baby, I was scared that my baby will not get breastmilk to suck when she is hungry*.” (Participant5).

One of the participant’s expressed that she wanted to discontinue breastfeeding because her lactation was still not established on the third day. She expressed that:

“*There was no breastmilk even on the third day after I gave birth. When I put my baby to the breast, there’s no milk when she sucks. I got tired at a point and wanted to stop putting my baby to breast*.” (Participant 11).

#### Positioning Baby to the Breast

According to participants, positioning was a major problem. They mentioned that they had a problem with positioning the baby correctly at the initial stages of breastfeeding. Although they were taught by the midwives severally they still couldn’t position their baby properly. A participant expressed that:

“*I had difficulty positioning my baby during breastfeeding even though I was taught at the hospital by the midwives and my mother also taught me when I came home*.” (Participant 12)

Another participant also intimated that;

“*The midwives in the hospital severally demonstrated and taught me how to position my baby correctly during breastfeeding but positioning my baby correctly was not easy for me but as time went on, I was able to position my baby correctly*.” (Participant 1)

#### Pain in the Nipple and Back

Study participants mentioned pain as a major problem when it comes to EBF. Many of the participants mentioned back pain as a result of poor posture during breastfeeding. They also said most first time mothers experience pain as they have cracked and sore nipple which was very painful when their baby is sucking. A participant expressed that:

“*It wasn’t easy for me because I had a cracked nipple so anytime my child wants to suck, in fact it wasn’t easy and it was very painful. I also experienced severe back pains due to the way I sit when I’m breastfeeding my baby*.” (Participant 1)

Another participant also said;

“*Few days after I started breastfeeding my child, my nipples became sore and this made breastfeeding very uncomfortable for me. When my baby cries and I want to breastfeed, it’s not easy but I had to because I didn’t want to give my child anything food or water*.” (Participant 8)

### Role of Family Support

Study participants said the role the family of a breastfeeding mother plays to encourage or discourage the first-time mothers from practicing exclusive breastfeeding. According to them, family relationship and support to mothers after childbirth helps improves their physical and mental health and restore confidence and decreases the mother’s stress level which helps mothers to exclusively breastfeed their baby.

#### Relationship With Family Members

Study participants mentioned good and close relationship with their family members helps in EFB practices. A participant expressed that she lives with her husband and mother in law, her parents and she has a good relationship with the family but because she shares a closer relationship with her mum and it gives her the strength to breastfeed. Other participants similarly expressed they shares a close relationship with their families. A participant said;

“*I live with my aunt and her husband. I have a good relationship with my aunt. She is the one who takes care of me and my baby.*” (Participant 3)

#### Relationship With Spouse

Participant mentioned the relationship they had with their husbands. Almost all of the participants live with their husbands, some of them said their husbands were working in other town but they have a good relationship with them. One of the participants expressed that;

“*I live with my husband. We have a good relationship, he was very supportive in my breastfeeding journey*.” (Participant 7)

#### Support From Family Members

Participants mentioned that family support is very crucial when it comes to the practice of exclusive breastfeeding. They intimated that support may also be effective in reducing the number of mothers who stopped breastfeeding earlier than the stipulated time. Many of the participants expressed that they received maximum physical and emotional support from their immediate family members. A participant said;

“*I received a lot of support because when I went back to my house after my child’s naming ceremony, my family knew that I was giving my child only breastmilk so they supported my decision and they don’t give my child water without my consent.*” (Participant 1)

However there were few of the participants who did not get any encouragement to continue exclusive breastfeeding. They expressed that some family members discourage them from practicing exclusive breastfeeding. One of the participant expressed that;

“*My mother in law and some relatives insisted that I must give my child water during her bath.*” (Participant 7)

### The Role of Society Influence

Study participants mentioned that the society plays a role when it comes to breastfeeding practices. They were of the view that, the society can encourage or discourage mothers to continue breastfeeding for the stipulated period for breastfeeding. The role of community members as well as support from health workers play a role when it comes to exclusive breastfeeding.

#### Support From Community Members

Study participants mentioned that the community members’ supports them by helping with their house chores and sometimes help them to hold their baby when they are busy and they offer them advice when needed. One participant expressed that:

“*My community members are friendly and good people, they give me good advice sometimes. They helped and supported during my child’s outdooring*.” (Participant 2)

However one of the participant expressed that the community people are not friendly. She intimated that;

“*This community people character is not good for me, their behavior is bad and they are not friendly.*” (Participant 3)

In terms of support with regards to practicing exclusive breastfeeding, many of the participants, intimated that, some of the community members discourages them by always telling them to give water to the child because of thirst and dry throat. A participant expressed that;

“*A woman asked me to give my child water but I said no. She and other community members around complained that it’s not good for the children because they gave their children water in the past and nothing happened to them.*” (Participant 12)

#### Support From Health Workers

Many of the participants expressed that they practiced exclusive breastfeeding because of the advice given to them by midwives and nurses after delivery and during their visits to the child welfare clinic. Participants believed that health workers are the ones who know what is good for our health, they have much knowledge on things so we trust what they say, and therefore we listen to their advice. One participant said;

“*I decided to practice exclusive breastfeeding because the nurses at the child welfare clinic always educate and encourage us to practice exclusive breastfeeding whenever we go for weighing.*” (Participant 6)

## Discussion

The primary aim of this study was to explore the psychosocial factors influencing exclusive breastfeeding among first-time mothers attending post-natal clinics in the Salaga Municipal Hospital of the Savannah Region. The findings highlight the multifaceted challenges faced by these mothers, emphasizing both individual struggles and the influence of familial, societal, and healthcare support systems.

### Individual Challenges and Coping Mechanisms

The study revealed that first-time mothers faced significant stress during the initiation and duration of breastfeeding. Lactation problems were a major challenge, with mothers struggling to establish lactation immediately after birth. Despite these challenges, support from midwives and relatives played a critical role in encouraging mothers to persevere. Participants expressed how advice on dietary practices and techniques to promote breastmilk production helped them overcome their initial fears and frustrations, eventually enabling successful breastfeeding. These findings align with prior studies that emphasize the importance of psycho-social support in overcoming lactation-related challenges [12–15]. Encouragement and guidance from midwives and family members, including dietary tips to promote milk production, proved instrumental in helping the mothers overcome this hurdle.

Positioning the baby to the breast was another reported difficulty. Many mothers, despite guidance from midwives, initially struggled with proper positioning. This difficulty often caused discomfort, cracked nipples, and back pain, leading some participants to consider discontinuing breastfeeding. However, with time and continued support, the mothers adapted, demonstrating resilience and a commitment to their infants’ wellbeing. These findings corroborate existing literature highlighting the need for targeted interventions addressing pain management and proper breastfeeding posture.

### Role of Family Support

Family and spousal support emerged as pivotal factors in promoting EBF among participants. Positive family relationships, particularly with female relatives such as mothers, aunts, and mothers-in-law, were instrumental in guiding first-time mothers. These relatives provided emotional and practical support, boosting the mothers’ confidence and reducing stress levels. This finding supports existing studies that identify close, experienced female relatives as influential in breastfeeding practices. However, some participants reported conflicting advice, particularly from mothers-in-law who encouraged giving water to infants, which could undermine EBF. This discrepancy highlights the complex dynamics within families that can either support or hinder EBF efforts [16–18].

Interestingly, spousal support was particularly noteworthy, with many mothers expressing gratitude for their partners’ encouragement. The involvement of spouses not only reinforced the mothers’ resolve to continue exclusive breastfeeding but also helped them resist external pressures from family members advocating for supplemental feeding, such as giving water during bath time. This finding contradicts previous studies in Ghana [19], where fathers often discouraged exclusive breastfeeding, but aligns with research from Chad [20], which highlights the positive impact of spousal support on successful breastfeeding practices.

### Influence of Community and Cultural Norms

The broader community also played a dual role in either encouraging or discouraging exclusive breastfeeding. On the one hand, community members often helped with household chores and provided moral support, enabling mothers to focus on breastfeeding. On the other hand, prevailing cultural norms and misconceptions frequently undermined exclusive breastfeeding practices. For instance, some community members believed that breastmilk alone was insufficient for infants and insisted on supplementing with water or formula. This finding resonates with studies in Nigeria [21], where concerns about the adequacy of breastmilk led to supplementation practices.

Nonetheless, the influence of elderly community members, who relied on past experiences to justify giving water to infants, further complicated the decision-making process for first-time mothers. However, the participants also acknowledged that a few community members actively promoted exclusive breastfeeding, underscoring the mixed societal attitudes toward the practice.

### Role of Healthcare Providers

Health workers played a critical role in educating and motivating mothers to practice exclusive breastfeeding. Participants highlighted the value of the advice they received during routine weighing appointments, which reinforced their confidence in continuing exclusive breastfeeding. Trust in the expertise of healthcare providers emerged as a key factor influencing adherence to recommended breastfeeding practices. This finding supports research conducted in Accra [22], which underscores the importance of maternal nutrition education provided by healthcare workers in promoting exclusive breastfeeding.

### Limitation

From the study, the small sample size may limit the generalizability of findings, while the purposive sampling method introduces potential selection bias. Reliance on self reported data raises concerns about recall and social desirability biases. Additionally, translation from the local language to English may have affected the interpretation of findings. The study’s exclusive focus on first-time mothers limits comparative insights with experienced mothers, and cultural norms were not deeply explored. Addressing these limitations in future research can enhance the depth and applicability of findings.

### Conclusion

The findings of this study demonstrate that exclusive breastfeeding among first-time mothers is influenced by a complex interplay of personal challenges, family dynamics, societal norms, and healthcare support. In Ghana, addressing lactation issues, pain management, and positioning challenges through tailored interventions can significantly improve breastfeeding experiences. Strengthening family and community support systems, alongside continuous education by healthcare providers, can further encourage adherence to exclusive breastfeeding practices. Therefore, fostering a supportive environment that addresses both individual and societal barriers, we can help more mothers successfully breastfeed exclusively for the recommended duration.
